# The Aphid-Transmitted *Turnip yellows virus* Differentially Affects Volatiles Emission and Subsequent Vector Behavior in Two *Brassicaceae* Plants

**DOI:** 10.3390/ijms19082316

**Published:** 2018-08-07

**Authors:** Patricia Claudel, Quentin Chesnais, Quentin Fouché, Célia Krieger, David Halter, Florent Bogaert, Sophie Meyer, Sylvaine Boissinot, Philippe Hugueney, Véronique Ziegler-Graff, Arnaud Ameline, Véronique Brault

**Affiliations:** 1 SVQV, Université de Strasbourg, INRA, 28 rue de Herrlisheim, 68000 Colmar, France; patricia.claudel@inra.fr (P.C.); david.halter67750@gmail.com (D.H.); Florent.Bogaert@inra.fr (F.B.); sophie.meyer@inra.fr (S.M.); sylvaine.boissinot@inra.fr (S.B.); philippe.hugueney@inra.fr (P.H.); 2 UMR CNRS 7058 EDYSAN, Université de Picardie Jules Verne, 80039 Amiens, France; chesnais.quentin.etud@gmail.com (Q.C.); quentin.fouche@gmail.com (Q.F.); Arnaud.Ameline@U-Picardie.Fr (A.A.); 3 Department of Entomology, University of California, Entomology Building, 900 University Ave., Riverside, CA 92521, USA; 4 CHU Lille, EA 7367—UTML—Unité de Taphonomie Médico-Légale, Université de Lille, 59000 Lille, France; 5 Institut de Biologie Moléculaire des Plantes, CNRS, Université de Strasbourg, 67000 Strasbourg, France; Celia.Krieger@Ibmp-Cnrs.Unistra.Fr (C.K.); Veronique.Ziegler-Graff@Ibmp-Cnrs.Unistra.Fr (V.Z.-G.)

**Keywords:** *Luteoviridae*, polerovirus, volatiles, aphids, *Brassicaceae*

## Abstract

Aphids are important pests which cause direct damage by feeding or indirect prejudice by transmitting plant viruses. Viruses are known to induce modifications of plant cues in ways that can alter vector behavior and virus transmission. In this work, we addressed whether the modifications induced by the aphid-transmitted *Turnip yellows virus* (TuYV) in the model plant *Arabidopsis thaliana* also apply to the cultivated plant *Camelina sativa*, both belonging to the *Brassicaceae* family. In most experiments, we observed a significant increase in the relative emission of volatiles from TuYV-infected plants. Moreover, due to plant size, the global amounts of volatiles emitted by *C. sativa* were higher than those released by *A. thaliana*. In addition, the volatiles released by TuYV-infected *C. sativa* attracted the TuYV vector *Myzus persicae* more efficiently than those emitted by non-infected plants. In contrast, no such preference was observed for *A. thaliana*. We propose that high amounts of volatiles rather than specific metabolites are responsible for aphid attraction to infected *C. sativa*. This study points out that the data obtained from the model pathosystem *A. thaliana*/TuYV cannot be straightforwardly extrapolated to a related plant species infected with the same virus.

## 1. Introduction

Insect-vectored plant viruses seriously affect important crops worldwide. Among insects, aphids are the most prevalent vectors which transmit a wide diversity of plant viruses [1,2]. Virus transmission by aphids relies on specific interactions between the virus, the plant, and the insect. Two major modes of virus transmission by aphids have been described based on the intimate interactions between the virus and the insect. The non-circulative, non-persistent viruses are acquired and inoculated during brief intracellular probing of aphids, and they rely on a transient and brief retention of virions at specific sites in the vector’s mouthparts, or in close proximity. In contrast, the circulative persistent viruses are acquired and inoculated during long phloem-sap ingestion phases and depend on virion endocytosis into the aphid cells [2,3,4,5,6,7,8]. In the latter mode of transmission, viruses may persist in the aphid’s body during the whole insect life, with (propagative mode) or without (non-propagative mode) replication. Upstream of the direct association between aphids and plant viruses, there is a complex process for the vector to select the host plant [9]. Aphid landing behavior can be driven by visual cues associated with external symptoms and volatiles emission that may attract insects onto the plant. Then, leaf surface properties and the first gustatory probing phases in the epidermal cells can also determine aphid acceptability of the host plant, or its rejection. The last phase that impacts aphid behavior is the composition of the sap that aphids ingest for sustained feeding [9]. There are compelling evidences that virus-induced alterations of plant cues may indirectly affect vector behavior, and hence virus transmission [10,11,12]. The hypothesis proposed by Mauck et al. [10] was that viruses that share a similar mode of transmission, and thus supposedly benefit from similar vector-plant interactions, would exhibit significant convergence in their effects on host phenotypes conducive to transmission.

Members in the *Luteoviridae* family, thereafter referred to as luteovirids, are circulative and non-propagative viruses, which are strictly transmitted by aphids. These phloem-limited viruses are transported through the aphid’s body by successive endocytosis mechanisms occurring at the intestinal and the accessory salivary gland levels [13,14] that require virus-specific receptors [15,16]. Many studies have documented that luteovirids induce modifications in their infected host plants that become more attractive to their aphid vectors in comparison to non-infected plants [17,18,19,20,21,22,23,24,25,26,27,28]. However, the indirect effect of luteovirid infection on aphids varies, depending on the combination of the plant, aphid and virus species studied [19,25,29]. A direct effect of luteovirid acquisition by aphids on their orientation preferences has also been reported after virus acquisition from plants, or from an artificial diet [22,24,29,30]. In particular, Ingwell et al. [30] showed that aphid vectors carrying *Barley yellow dwarf virus* (BYDV, *Luteoviridae* family) after an artificial acquisition of the purified virus were attracted to healthy wheat plants, whereas nonviruliferous aphids preferred odor cues from infected plants.

Although viral-induced plant modifications on aphids have been widely observed, the plant components responsible for the aphid behavior modifications are largely unknown. Aphid settling preference for luteovirid-infected plants can be due to virus-induced changes in sap quality such as amino acids and carbohydrate contents [31,32,33]. Plant volatile organic compounds (VOC) can directly impact on aphid alighting behavior, and aphids were shown to prefer luteovirid-infected over non-infected plants in the absence of physical contact and visual cues [21,22,26,28,34]. Aphid orientation and settlement were correlated with elevated amount of volatiles emitted by luteovirid-infected plants [20,25,27]. The VOC profile in the headspace of luteovirid-infected plants differed mostly in terms of the metabolites concentration and to a lesser extent, in the metabolites composition when compared to non-infected plants [20,23,27].

Our goal was to compare the impact of *Turnip yellows virus* (TuYV, *Polerovirus* genus, *Luteoviridae* family) on the volatile emission profiles and subsequent vector behavior on the model plant *Arabidopsis thaliana*, and on a genetically related cultivated plant, *Camelina sativa*, which are both in the *Brassicaceae* family. *C. sativa* was cultivated in Europe as an important oilseed crop for many centuries, and it has regained interest as an emerging biofuel crop [35]. We used a non-targeted approach to analyze the volatile composition of infected and non-infected plants. The relative volatile amount emitted by TuYV-infected plants was always higher compared to mock-inoculated plants for *C. sativa*, whereas no such consistency was observed for *A. thaliana*. Moreover, the global volatile emission from *C. sativa* individual plant was higher than *A. thaliana* plants regardless of the virus infection. The effects of volatiles on *M. persicae* (vector of TuYV) were addressed by performing choice tests. Interestingly, *M. persicae* was more attracted by TuYV-infected *C. sativa* than by non-infected plants, while no preference was observed for *A. thaliana*. Our study reveals that the impact of virus infection on volatile emission and subsequent aphid behavior is pathosystem-specific, and that the data obtained with a model plant such as *A. thaliana* cannot be straightly transposed to closely related species.

## 2. Results

### 2.1. Differential Headspace Volatile Organic Compounds (VOC) Emission from TuYV-Infected C. sativa and A. thaliana

In order to exclude any TuYV effect on the VOC emission from infected plants that could be linked to a specific physiological status of the plant and that could not be reproduced under different environmental conditions, plants used for the VOC analyses were grown under different light sources (fluorescent or light-emitted diode (LED) lamps). Plants were inoculated by infiltration of agrobacteria containing a binary plasmid with the TuYV full-length sequence, or bearing an empty binary plasmid (mock-inoculated plants). Three sets of inoculation of *C. sativa* and *A. thaliana* were performed. In the three experiments performed on *C. sativa*, TuYV-inoculated plants developed yellowing symptoms and exhibited reduced growth compared to mock-inoculated plants (Figure S1a). The symptomatic TuYV-inoculated *C. sativa* were used for volatile sampling, and their infectious status was confirmed after volatile collection by double-antibody sandwich enzyme-linked immunosorbent assay (DAS-ELISA). TuYV-infected *A. thaliana* exhibited mild reddening symptoms in two experiments out of three when plants were grown under the LED lamps (Figure S1b). Since the symptoms were only apparent on the lower leaf surface of old leaves, and could be attributed to an additional abiotic stress response by the plants, virus infection of *A. thaliana* was always assayed by DAS-ELISA three weeks post-inoculation. The volatiles were collected one week after the ELISA test to avoid the potential effect of plant damage on volatiles emission, due to the sampling for the ELISA test. In the three experiments performed, all TuYV-infected *A. thaliana* showed a reduced weight compared to non-infected plants (Figure S1b). Globally, these observations showed that symptoms expression on *C. sativa* is not influenced by light growth conditions, in contrast to *A. thaliana*, for which the mild reddening symptoms appeared only when plants were grown under LED lamps (Figure S1). The headspace of TuYV-infected or non-infected *C. sativa* and *A. thaliana* were collected three to four weeks post-agroinoculation from whole plants during 24 h, and analyzed by gas-chromatography mass spectrometry (GC-MS) with a non-targeted approach.

**Figure 1:**
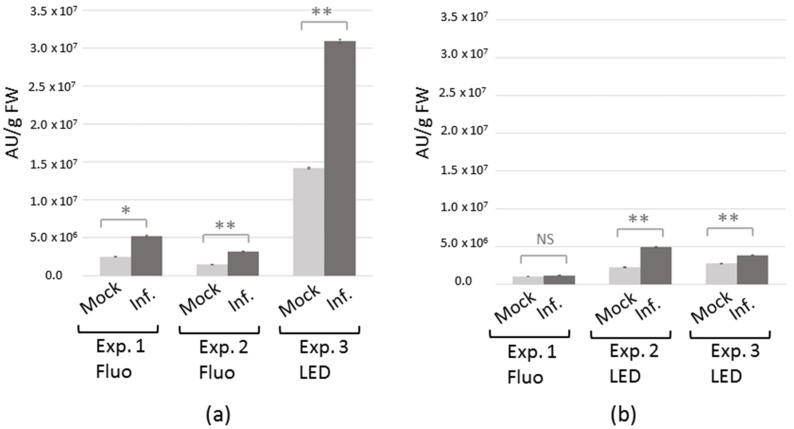
Impact of *Turnip yellows virus* (TuYV) infection on the relative emission of volatiles collected from entire *C. sativa* (**a**) and *A. thaliana* (**b**) plants. Bars (black for TuYV-infected plants and grey for mock-inoculated plants) represent the mean total chromatogram area ± SE (3 experiments with *n* = 5 replicates of two plants for *C. sativa*; three experiments with *n* = 5 replicates of seven plants for *A. thaliana*), expressed in area units (AU) per g of fresh weight (FW). The light source used to grow plants is indicated (Fluo = cool-white fluorescent lights and LED = Light-emitted diode lamps). Statistical significance of differences was tested using the Wilcoxon test. NS = not significant = *p*-value > 0.05; * = 0.01 < *p*-value ≤ 0.05; ** = *p*-value ≤ 0.01.

In order to evaluate the effect of TuYV infection, VOC emissions of infected and mock-inoculated plants were compared in three independent experiments. Experiments involved five replicates of two *C. sativa* plants for each condition (infected or mock-inoculated) and five replicates of seven *A. thaliana* plants for each condition. For each group of plants, the total chromatogram area representing the global amount of VOC emitted was calculated by adding the areas of all the peaks from the metabolic profile. A total number of 381 metabolites were detected for *C. sativa* and 279 for *A. thaliana*. Interestingly, we observed that TuYV infection significantly enhanced the relative VOC emission in both *C. sativa* (Exp. 1 *p*-value = 0.032, Exp. 2 *p*-value = 0.008, Exp. 3 *p*-value = 0.008, Figure 1a) and *A. thaliana* (Exp. 2 *p*-value = 0.008, Exp. 3 *p*-value = 0.008, Figure 1b), except for one experiment with *A. thaliana* (Exp. 1 *p*-value = 0.548, Figure 1b). In this experiment (Exp. 1, Figure 1b) *A. thaliana* plants were grown under fluorescent lamps. In these specific environmental conditions, infected *A. thaliana* had no visible symptoms, and the difference in plant size between infected and non-infected plants was not as pronounced as in the other conditions (Figure S1). We also observed a higher global relative VOC emission in *C. sativa* from experiment 3 (Figure 1a), which could be due to the light conditions (LED), to an earlier virus inoculation in this experiment (two weeks instead of three weeks after planting) resulting in reduced plant size (Figure S1), or to a combination of both.

**Figure 2:**
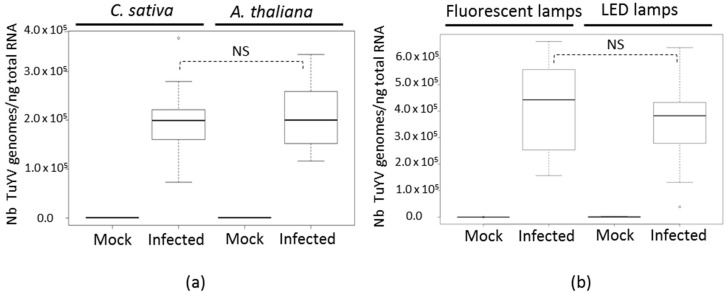
Analysis of TuYV accumulation by quantitative real-time reverse transcriptase-polymerase chain reaction (RT-PCR) in non-infected and infected *C. sativa* and *A. thaliana*. (**a**) Virus titer was analyzed at 20 (*C. sativa*, *n* = 3 mock-inoculated and *n* = 11 TuYV-infected plants) and 19 (*A. thaliana*, *n* = 3 mock-inoculated and *n* = 6 TuYV-infected plants) days post-inoculation. In this assay, plants were grown under fluorescent lights. (**b**) Virus titer was analyzed four weeks post-inoculation in *A. thaliana* cultivated under fluorescent lamps (*n* = 7 mock-inoculated and *n* = 12 TuYV-infected plants) or LED lamps (*n* = 3 mock-inoculated and *n* = 10 TuYV-infected plants). Error bars represent means ± SE for the replicates. Statistical significance of the differences between infected plants was tested using a Mann-Whitney U test. NS = not significant = *p*-value > 0.05.

To address whether in similar plant growth conditions, the differences in the volatile emission profiles observed could be related to virus accumulation, TuYV genomes were quantified by real-time reverse transcription (RT)-polymerase chain reaction (PCR) three weeks post-inoculation in plants grown under fluorescent lamps (as in Exp. 1 and 2 for *C. sativa* and Exp. 1 for *A. thaliana*, Figure 1). As shown in Figure 2a, TuYV genomes accumulated at a similar level (*p*-value = 0.884) in infected *C. sativa* (2.03 × 10^5^ genome copies per ng of RNA) and in *A. thaliana* (2.10 × 10^5^ genome copies per ng of RNA). This shows that in these growth conditions, the higher amount of volatiles emitted by infected *C. sativa* compared to the control plants (Exp. 1 and 2 for *C. sativa*, Figure 1a) was not due to a higher virus accumulation when compared to infected *A. thaliana*. Since the light conditions could also potentially impact virus accumulation in the same plant species, we then compared the TuYV genome accumulation in infected *A. thaliana* grown under fluorescent lights (Exp. 1 for *A. thaliana*, Figure 1b) or LED lamps (Exp. 2 and 3 for *A. thaliana*, Figure 1b). As shown in Figure 2b, the level of TuYV accumulation in infected *A. thaliana* grown under LED lamps (3.48 × 10^5^ genome copies per ng of RNA) was not higher (*p*-value = 0.381) than the one from plants which were developed under fluorescent lamps (4.14 × 10^5^ genome copies per ng of RNA). Again, these results demonstrated that the amount of volatiles emitted by infected plants was not correlated to the virus titer.

**Figure 3:**
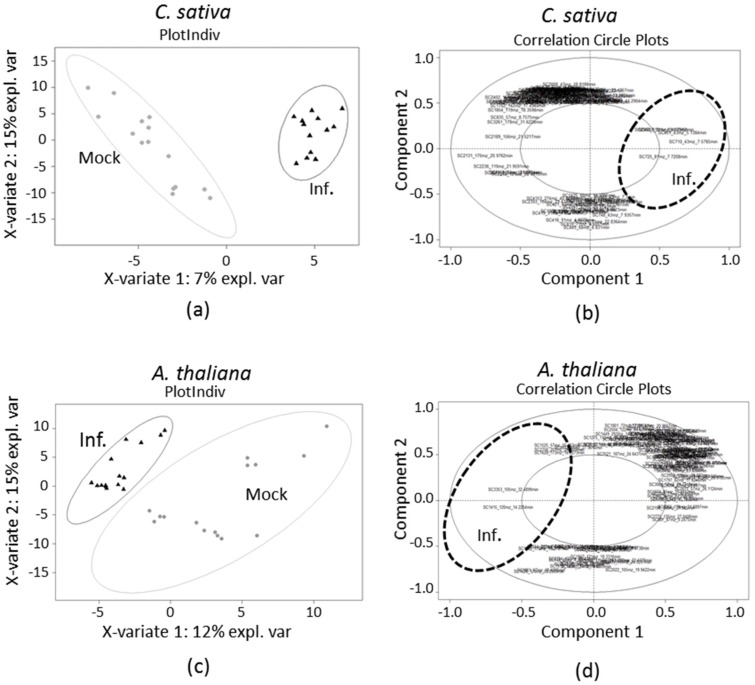
Multivariate analysis using partial least-square discriminant analysis (PLS-DA) of volatile organic compounds (VOC) profiles emitted from *C. sativa* (**a**,**b**) and *A. thaliana* (**c**,**d**) TuYV-infected and non-infected plants. (**a**,**c**): PLS-DA sample plots. Each symbol represents two plants for *C. sativa* and seven plants for *A. thaliana*. Black triangles stand for TuYV-infected plants and grey circles for mock-inoculated plants. Confidence ellipses for each group are plotted to highlight the strength of the discrimination (confidence level set to 95%). (**b**,**d**): PLS-DA correlation circle plots. The variables (volatiles) are represented through their projections onto the plane defined by the first two dimensions on the correlation circle of radius 1. A threshold of 0.5 is set to remove weaker correlations and to plot only volatiles with major importance. Strongly correlated variables are projected in the same direction from the origin. The distance from the origin is correlated to the strength of the association. The dashed ellipses tentatively highlight the metabolites which are the most correlated with infection of *C. sativa* (**b**) and *A. thaliana* (**c**).

We then focused on a qualitative analysis of the metabolite composition of the VOC blends that were emitted by infected and non-infected plants. Supervised multivariate analysis is appropriate for distinguishing differences between VOC blends emitted by infected or non-infected plants [36]. In this regard, the data sets were analyzed with partial least-square discriminant analysis (PLS-DA). From the sample plots (Figure 3a,c), we observed a separation between infected or non-infected plants for both species. Concerning *C. sativa*, positive score values were correlated with infected plants, while mock-inoculated plants were correlated with negative values. On the contrary, for *A. thaliana* positive score values were correlated with mock-inoculated plants while infected plants were correlated with negative values. The separation between the two groups (infected versus mock-inoculated) was less pronounced for *A. thaliana*, suggesting that infected and non-infected plant volatile profiles share more similarities than those from infected and non-infected *C. sativa*. Moreover, the dispersion within the non-infected groups was increased for *A. thaliana* compared to *C. sativa*, suggesting a higher variability in the volatile profiles of non-infected *A. thaliana* (Figure 3a,c).

From the correlation circle plots showing the projection of the volatiles emitted by the plants on the principal axes obtained by PLS-DA, it was possible to visualize the volatiles that were the most strongly linked with infected or mock-inoculated plants (Figure 3b,d). Volatiles that were positively correlated with a class of plant species are projected in the same direction from the origin. These plots revealed that few volatiles were strongly correlated with infected plants in *C. sativa* (positive scores values) (Figure 3b) and in *A. thaliana* (negative scores values) (Figure 3d). The major part of the volatiles tended to be more closely associated with mock-inoculated plants for both plant species.

### 2.2. Identification of Volatiles Discriminating Infected from Non-Infected C. sativa and A. thaliana

Volatiles that discriminate mock-inoculated from infected plants were selected from the values of regression coefficients, VIP (variable importance in projection) indices provided by PLS-DA analysis and *p*-values obtained by ANOVA. According to the criterion of VIP > 1.0, correlation > |0.5| and *p*-value < 0.05, a total of ten metabolites for *C. sativa* and six for *A. thaliana* were identified as factors contributing to the discrimination between infected and non-infected plants (Table 1). One volatile, the 6-methyl-5-hepten-2-one, was overexpressed in infected *C. sativa* plants, while the other volatiles, including four sesquiterpenes (*cis*-thujopsene, cyclosativene, α-barbatene and β-alaskene), were more abundant in mock-inoculated *C. sativa* plants. Regarding *A. thaliana* plants, six compounds could discriminate infected from non-infected plants (Table 1). Among them, methyl salicylate was over-emitted in infected plants, whereas 1-phenoxybenzene, 4-*tert*-octylphenol, and methyl 2-ethylhexanoate were more released by mock-inoculated plants. β-selinene and 3-methylbenzamide were only detected in non-infected plants.

### 2.3. The Non-Viruliferous Myzus persicae are Attracted by TuYV-Infected C. sativa but Not by TuYV-Infected A. thaliana

Preference tests were performed with both plant species by giving non-viruliferous *M. persicae* the choice between plants that were inoculated with viruliferous or non-viruliferous aphids, or to empty pots filled with soil (blank) in different combinations. Aphids were allowed to move freely in any direction with no visual cues or physical contact with the plant. One hour after release, aphids were counted under each plant or empty pot, and the aphids still walking in the arena and that were not located in the target areas below the leaflets or pots were considered as non-responding. As mentioned in Eigenbrode et al. [20], the close distance between the leaves and the aphids (about 2 mm) exposes the insects to the headspace volatiles of each plant and minimizes the potential influence of mixed volatiles within the arena. *C. sativa* and *A. thaliana* were used for the choice test three weeks post-inoculation. The TuYV-inoculated plants developed symptoms but their infection status was however, controlled by performing a DAS-ELISA test at the end of the experiment.

**Figure 4:**
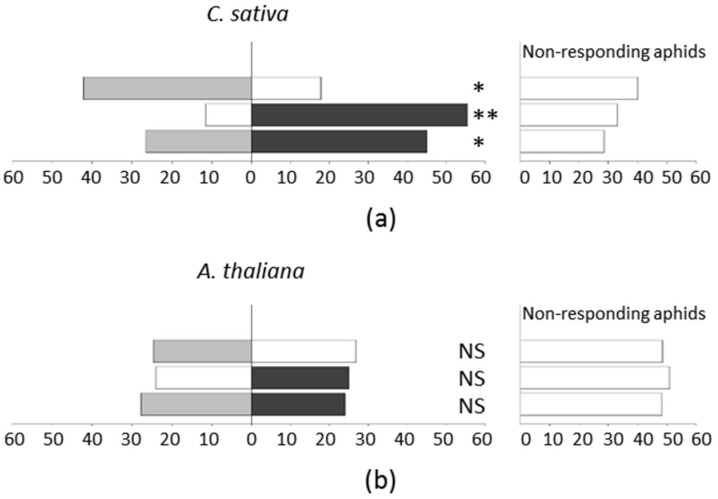
Distribution of apterous *M. persicae* in the darkened bioassay arena. (**a**) *C. sativa*; (**b**) *A. thaliana*. Mock-inoculated plants (grey bars) and TuYV-infected plants (black bars) were tested versus either the blank (white bars) or the opposite modality. The bars on the left represent the percentage of responding aphids that were arrested under either a plant or an empty pot (Blank). The bars on the right represent the percentage of non-responding aphids still walking in the arena. Asterisks indicate a significant difference (* *p*-value < 0.05; ** *p*-value < 0.01) in the distribution of aphids between the two sides of the darkened arena. NS, not significant.

For all of the different combinations tested, the mean percentages of non-responding insects ranged from 28.6% to 40.1% for *C. sativa* (Figure 4a), and from 42.8% to 53.8% for *A. thaliana* (Figure 4b) and they were not significantly different between the combinations (Kruskal–Wallis: *H* = 3.742, *df* = 2, *p*-value = 0.154 for *A. thaliana* and *H* = 3.515, *df* = 2, *p*-value = 0.172 for *C. sativa*). Only the responding aphids were considered in the subsequent analyses. When tested against a blank, *M. persicae* displayed a significant preference for *C. sativa*, whatever its infectious status (Wilcoxon: *Z* = 55, *p*-value = 0.006 for TuYV-infected *C. sativa*; Wilcoxon test: Z = 52, *p*-value = 0.014 for non-infected *C. sativa*) (Figure 4a). In contrast, *M. persicae* did not exhibit any preference between an empty pot and *A. thaliana*, regardless of the infection status (Wilcoxon: Z = 36.5, *p*-value = 0.384 for TuYV-infected *A. thaliana*; Wilcoxon test: Z = 14.5, *p*-value = 0.202 for non-infected *A. thaliana*) (Figure 4b). Interestingly, *M. persicae* displayed a significant preference for TuYV-infected *C. sativa* versus non-inoculated plants (Wilcoxon: *Z* = 6, *p*-value = 0.032) (Figure 4a), whereas no such preference was observed for *A. thaliana* (Wilcoxon: Z = 48, *p*-value = 0.503) (Figure 4b). An independent experiment was performed using *C. sativa* plants that were non-infected or inoculated by agroinoculation, or with viruliferous aphids. A similar preference of aphids for infected plants was observed whatever the mode of virus inoculation, discarding a potential effect of the virus delivery method on the aphid preference (Figure S3).

## 3. Discussion

There is a growing body of literature about viruses’ manipulation of insects and plants, showing that viruses can interfere at all steps of insect-plant interactions to enhance transmission [10,12]. Virus-induced plant modifications may impact aphid attraction and landing on the plant, through alteration of plant volatile emission. The plant changes caused by the virus can also influence virus uptake by aphids by affecting the metabolite content of plant cells, and therefore the aphid feeding behavior. Viral factors that influence vector behavior are starting to be identified, such as the satellite virus of *Tomato yellow leaf curl China virus* and the 2b protein of *Cucumber mosaic virus*, which modify phytohormones pathways [37,38,39,40]. Depending on the mode of virus transmission (circulative and non-circulative modes), it is generally admitted that the virus will induce distinct modifications in the plant, since virus acquisition and inoculation by aphids does not rely on the same parameters (brief probes in epidermal cells for non-circulative viruses and long phloem-feeding phases for circulative viruses). Even if the hypothesis of “virus manipulation” is supported by numerous studies on different pathosystems (reviewed in Mauck et al. [12]), the virus’ impact on aphids has been shown to vary depending on the virus isolate, the aphid species, and the virus transmission efficiency by the aphid species. This precludes any generalization of the plant manipulation concept to viruses belonging to the same family, or even to the same genus. Moreover, manipulation of host phenotype is species-specific and can be limited to certain genotypes within a species. This suggests that plant viruses cannot evolve manipulative functions that induce a transmission-conducive phenotype in all possible hosts (reviewed in Mauck et al. [12]).

In this paper, we addressed whether the phloem-limited and strictly aphid-transmitted TuYV (circulative and non-propagative mode) could impact the volatile emission of two genetically closely related plant species belonging to the *Brassicaceae* family, with direct consequences on aphid behavior [41].

We observed a propensity of TuYV to increase the relative amounts of VOC emitted by TuYV-infected plants of both plant species *C. sativa* and *A. thaliana*. The significant and reproducible effect of the virus infection in *C. sativa* was independent of the light conditions, whereas VOC emission from infected *A. thaliana* varied depending on the light source used to grow the plants. A significantly higher relative VOC emission was emitted by infected *A. thaliana* in two experiments out of three when plants were grown under LED lamps. These different experiments highlighted the crucial impact of abiotic factors as light on plant VOC emission [42]. Interestingly, we also observed that the differences in the relative VOC emission from infected plants between the various environmental conditions were not correlated to virus accumulation in plants but were always associated with symptom development on infected plants.

Due to the larger size of *C. sativa* compared to *A. thaliana* (Figure S1), the global VOC emission per plant was always higher in *C. sativa* compared to *A. thaliana*, whatever their infectious status (Figure S2). It is likely that the high VOC emission from both infected and non-infected *C. sativa* is responsible for the attraction of *M. persicae* to these plants when they were exposed simultaneously to empty pots. Indeed, in a similar experimental setting, aphids did not show preference for *A. thaliana* plants when exposed simultaneously to empty pots. Interestingly, the choice tests revealed that *M. persicae* was preferentially attracted to TuYV-infected *C. sativa* but not to TuYV-infected *A. thaliana* when *M. persicae* was exposed without visual or physical cues to both infected and mock-inoculated plants of the same species. It is therefore tempting to conclude that either the higher amount of VOC blend emitted by TuYV-infected *C. sativa* compared to mock-inoculated plants, or the specific compounds in the blend of infected *C. sativa* attract *M. persicae*. The qualitative VOC analyses identified 10 metabolites that were differentially emitted between infected or non-infected *C. sativa*. Among these metabolites, only 6-methyl-5 hepten-2-one was preferentially emitted by infected plants. This plant compound has been previously shown to repel aphids from aphid-infested plants [43], and is supposed to be a specific indicator of the presence of aphids for parasitoids [44,45], albeit in different contexts involving different aphid and plant species. It is therefore unlikely that this unique compound explains the aphid attraction towards infected *C. sativa*. The observed aphid preference for infected *C. sativa* may therefore be the result of the global TuYV-induced increase in VOC emission, which probably overcomes the potential repellent effect of the 6-methyl-5 hepten-2-one. We also found six metabolites that differentiated infected from non-infected *A. thaliana*, and among them, methyl salicylate was the only compound that was over-emitted by the infected plants. This compound is an herbivore-induced plant volatile that has been shown to have an aphid repellent effect [46], and it attracts aphid natural enemies [47,48]. Interestingly, methyl salicylate was also produced by plants infected with the *Tobacco mosaic virus*, and was identified as an airborne signal to confer virus resistance to neighboring non-infected plants [49]. Considering that *M. persicae* did not display any preference for infected or non-infected *A. thaliana* plants, it is likely that these six compounds are not perceived by the aphids, at least at the concentrations emitted by the plants, or that they have no effect on aphids.

Overall, to explain aphid behavior, our data are more in favor of the total amount of VOC emitted by infected plants having a predominant role, rather than the specific composition of the blend. This work reinforces the hypothesis that the volume of the VOC blend may impact aphid attraction more than the nature of specific metabolites [22,23]. However, except for Rajabaskar et al. [25] who reported a difference in VOC emission and aphid selection between potatoes varieties infected with PLRV, and Jimenez-Martinez et al. [21], who worked with a transgenic wheat resistant cultivar, this is the first report showing that VOC emission may vary between two species belonging to the same family and being infected with the same virus. Consequently, TuYV impact on the plant volatile profile cannot be generalized from experiments performed on model plants like *A. thaliana*. Moreover, if we consider the aphid preference for infected *C. sativa* over healthy plants, TuYV infection of this plant species would be conducive to transmission. However, no such conclusion can be drawn for *A. thaliana*. Consequently, plant manipulation by TuYV would be species-specific and cannot be generalized to all plant species.

This work should be pursued by analyzing the effect of VOC emission on different aphid morphs including winged aphids since it has been shown that aphid development may influence aphid responsiveness to VOC [50,51]. Moreover, recent studies have shown that once the virus is acquired by the aphids, viruliferous aphid behavior is affected: the preference of non-viruliferous aphids for infected plants is reversed [24,29], and viruliferous aphids tend to react differently to host plant volatiles [22]. In this regard, it will be of particular interest to analyze *M. persicae* behavior after the acquisition of TuYV.

This study highlights the relevance of introducing several plant species when analyzing virus-induced plant effects on aphid behavior. Indeed, the observed contrasted aphid behavior towards VOC emitted by different plant species may have a true impact in nature. Although preferential colonization of virus-infected plants by aphids has been attributed to the yellowing of infected tissues, which becomes more visually attractive to aphids [52,53], the VOC detected in close proximity to the leaf surface may impact aphid behavior. In particular, the leaf surface exploration and the probing of subepidermal tissues for walking aphids (apterous aphids) can be affected, since these aphids use volatile cues to locate a proper host [51]. During this stage, aphids exhibit antennal waving on the plant surface, evidently facilitating the detection of odors in the boundary layer close to the plant surface. In this work, the observed contrasted aphid behavior may impact rates of virus spread with implication in virus-disease management [54,55]. In an epidemiological scheme, wild plants or weeds can play a role of virus reservoir and can impact virus propagation. In particular, it is conceivable that a most preferred or attractive infected wild plant will divert the vector from the crop but the situation can also occur in reverse. Current management strategies against vector-borne virus diseases rely mostly on eliminating virus vectors by chemical treatments to reduce virus outbreaks [1]. Understanding how aphids locate virus-infected host plants can result in the control of populations by innovative approaches such as the selection of cultivars with repellent properties.

## 4. Materials and Methods

### 4.1. Plant Growth and Aphid Rearing

Seeds of camelina (*Camelina sativa* cv. “Celine”) were provided by the Technical Institute in Agronomy Terres Inovia (Paris, France). For the dual choice assay, *C. sativa* and *A. thaliana* (Col0 ecotype) were grown in a growth chamber under 20 ± 1 °C and a 16 h photoperiod under fluorescent lamps. For all the other experiments performed with both *C. sativa* and *A. thaliana*, plants were grown under a 10 h photoperiod. Growth chambers were equipped with either (i) cool-white fluorescent lights yielding a light intensity of 250 to 300 μmol m^−2^ s^−1^ (300–800 nm) (*C. sativa,* experiments 1 and 2; *A. thaliana* experiment 1); (ii) LED lamps yielding a light intensity of 180 µmol m^−2^ s^−1^ (450–670 nm) (*C. sativa,* experiment 3; *A. thaliana*, experiments 2 and 3). Plants were inoculated three to four weeks after sowing, except in experiment 3 for *C. sativa* in which plants were inoculated two weeks post-sowing. The *Myzus persicae* (Sulzer) (Hemiptera: Aphididae) clone used in the choice test assay was established from one parthenogenetic female collected in a potato field and reared on rapeseed (*Brassica napus* cv. “Adriana”) (*Brassicaceae*) in a growth chamber under 20 ± 1 °C, and 16 h photoperiod [19].

### 4.2. Plant Inoculation with TuYV by Agroinoculation or Viruliferous Aphids and Viral Detection

*Agrobacterium tumefaciens* GV3101 containing the plasmid pBIN harboring the complete TuYV genome [56] or the empty vector were grown to an OD_600nm_ of 0.5 before being agroinfiltrated into three-week old *C. sativa* or *A. thaliana* [57]. Alternately, *C. sativa* and *A. thaliana* were inoculated using viruliferous aphids as described in Chesnais et al. [19].

TuYV was detected in non-inoculated leaves of *C. sativa* or *A. thaliana* by DAS-ELISA with a TuYV-specific polyclonal antiserum (Loewe, Sauerlach, Germany) [58].

### 4.3. RNA Preparation and Quantitative Reverse Transcription-PCR

Total RNA from leaf tissues were extracted using Trizol reagent following the manufacturer’s recommendations and treated with DNase I to remove any DNA contamination. RNA extraction was done from plants grown under fluorescent and LED lamps. One µg of total RNA from *A. thaliana* and 1.5 µg from *C. sativa* was denatured and reversed-transcribed with 0.2 µg of random hexamer (ThermoFisher Scientific, Waltham, MA, USA) primers, 1 mM of dNTP, 4 µL of 5× reaction buffer, 1 µL of RevertAid^TM^ H minus Reverse Transcriptase (ThermoFischer Scientific), in a final volume of 20 µL by incubating for 1 h at 42 °C. PCR were performed using SYBR green I Master reagent according to the manufacturer’s recommendations and Light Cycler 480 apparatus (Roche Life Science, Penzberd, Germany) with the TuYV-specific primers, as described in Reinbold et al. [59]. The primers amplified a viral sequence from the ORF3 encoding the major coat protein and present on both genomic and subgenomic viral RNAs. Reactions were performed with 5 µL of Master Mix, 2.5 µM of each forward and reverse primers, and 1 µL of the two-fold diluted cDNA in a final volume of 10 µL. PCR runs were performed as follows: initial denaturation at 95 °C for 5 min; 45 amplification cycles at 95 °C for 10 s, 60 °C for 15 s, 72 °C for 15 s; real-time fluorescence measuring and denaturation at 95 °C for 5 s. Serial 10-fold dilutions of pBS plasmid encoding the complete TuYV genome were used to establish standard curves. Plasmid copy numbers were calculated as follow: (DNA in ng) × Avogadro number/(Plasmid size in bp × 10^9^ × 650 ng/nmol). The normality of the data distribution was tested for each dataset using the Shapiro-Wilk test and a Mann-Whitney U test was then applied to the data.

### 4.4. Dual-Choice Assays and Statistical Analysis

Aphid preference tests for TuYV-infected or mock-inoculated plants was adapted from Eigenbrode et al. [20]. *C. sativa* and *A. thaliana* were used three weeks after inoculation with viruliferous or non-viruliferous aphids [19]. The plants were grown under fluorescent lamps. The bioassay was carried out in an environmentally controlled room at 22 ± 1 °C, and 35 ± 10% relative humidity in the dark to eliminate any possible visual cues. The apparatus consisted of an arena made of a Plexiglas cylinder of 14 cm diameter with a floor made of a double screen (mesh size ca. 0.5 mm). Whole plants (for *A. thaliana*), leaflets still attached to the plants (for *C. sativa*) or empty pots (Blank) were inserted opposite one another and ca. 5 mm above the screen. A Petri dish was placed beneath the screen were twenty-five apterous aphids previously starved for 1 h were placed. The double screen ensured that aphid mouthparts could not reach the leaflets. One hour after introduction, aphids positioned on either side of the mesh were counted. Aphids located on the Petri dish were considered as non-responding. To avoid any possible physical bias, a rotation of 180° of the chamber was effected between replications. The chamber was cleaned with TFD4 detergent between each replicate. Ten replicates were made per treatment. For each replicate, new batches of aphids and new plants were used. A Kruskal–Wallis test was performed to analyze the effect of treatment on the number of non-responding insects in the darkened arena bioassay. For each treatment in the dual choice experiments, the distribution of the responding aphids was analyzed using a Wilcoxon test for paired samples.

### 4.5. Headspace Collection and Analysis

Volatile compounds emitted by infected and mock-inoculated plants were extracted three to four weeks after agroinoculation by Headspace Sorptive Extraction (HSSE). This technique is a solvent-free extraction method which is not affected by matrix effects, and it is highly sensitive [60]. Three independent experiments were performed for both *C. sativa* and *A. thaliana.* Each experiment involved five replicates of two plants of *C. sativa* for each condition (i.e., five groups of two infected plants and five groups of two control plants). For *A. thaliana*, each experiment involved five groups of seven infected plants and the same number of control plants. VOC were collected from the headspace of entire plants after carefully removing them from potting soil and washing the roots with distilled water to eliminate soil residues. The plants were then placed in a volatile collection device consisting of 1 L glass jars closed with a glass lid (Le Parfait^®^, Villeurbanne, France). Volatiles were captured on two polydimethylsiloxane stir bars (Twister^®^, Gerstel GmbH & Co.KG, Mülheim an der Ruhr, Germany) positioned inside the glass jar and held magnetically (Figure S4). The jars were then placed on an IKA KS260 orbital shaker stirring at 100 rev/min and VOC sampling was carried out at 22 °C for 24 h. Prior to use, the stir bars were submitted to a helium flow for 20 min at 220 °C. After sampling, both stir bars were placed together in a single glass desorption liner to be simultaneously thermally desorbed. The desorbed compounds were analyzed by thermal desorption–gas chromatography–mass spectrometry (TD-GC-MS). TD-GC-MS analysis was performed with a thermal desorption unit (TDU) equipped with a MPS2 auto-sampler and a cryostatic cooled CIS-4 programmed temperature vaporization (PTV) inlet (Gerstel) installed on an Agilent 7890 B gas chromatograph with a 5977 B mass-selective detector (Agilent Technologies, Santa Clara, CA, USA). The stir bars were thermally desorbed by programming the TDU from 30 °C (held for 0.2 min) to 250 °C (held for 5 min) at 60 °C/min with 50 mL/min desorption flow. Desorbed compounds were focused at −20 °C onto a baffled glass liner in the cooled PTV inlet for subsequent GC–MS analysis. After desorption, the PTV inlet was programmed from −20 °C to 300 °C (held for GC run time) at 10 °C/s to inject trapped compounds onto the analytical column. The injection was performed in the solvent vent mode with a split vent flow of 20 mL/min. A HP-5MS capillary column (30 m, 0.25 mm internal diameter, 0.25 µm film thickness, Agilent Technologies) was used for separation. The chromatographic program was set at 40 °C for 10 min, raised to 300 °C at a rate of 4 °C/min and held at the final temperature for 10 min. A constant helium flow of 1 mL/min was used. The temperatures for MS transfer line and ion source were 270 °C and 230 °C, respectively. Ionization voltage was 70 eV. Full scan mode was used for acquiring the data within the mass-to-charge ratio (*m*/*z*) mass range of 30–300 atomic mass units (a.m.u.). All data were recorded using MassHunter GCMS Data Acquisition B.07.04.2260. Blank runs using an empty glass tube were performed before and after each batch.

### 4.6. Data Processing and Multivariate Analysis

Chromatogram files were deconvoluted and converted to ELU format using the AMDIS Mass Spectrometry software, with the resolution and sensitivity set to medium. Chromatograms were then aligned and integrated using Spectconnect (http://spectconnect.mit.edu). Metabolites were generated by SpectConnect as SCxxx_yyymz_zzzmin, where xxx is the scan number, yyy the mass-to-charge ratio (*m*/*z*), and zzz the retention time in minutes. All metabolites found in the blank run, or potentially originating from the Twister^®^ or from the column bleeding, were removed from analysis. After removal of these contaminant metabolites, the integrated signal (IS) matrix from Spectconnect was standardized by the mass of each sample. Metabolites were identified by comparison to the NIST 14 standard reference database. Confirmation of metabolite identification was done by using standards when available. A Wilcoxon test was performed to analyze the differences in the relative abundance of VOC per gram of fresh weight emitted from infected or mock-inoculated *C. sativa* and *A. thaliana* plants. GC-MS data were processed with partial least squares discriminant analysis (PLS-DA) using the mixOmics R package. Metabolites with variable importance in the projection (VIP) > 1.0, regression coefficient (r) > |0.5| and *p*-value < 0.05 were considered as metabolites that could discriminate plant groups with different volatile profiles.

## Figures and Tables

**Table 1:** Volatiles differentially emitted from *C. sativa* and *A. thaliana* TuYV-infected and non-infected plants.

Plant Species	Metabolites ^1^	Identification	VIP	Correlation	*p*-Value
***C. sativa***	SC2131_170mz_20.9762 min	1-Phenoxybenzene	3.228	−0.754	0.00
	SC2236_119mz_21.9591 min	*cis* -Thujopsene	2.585	−0.636	0.04 × 10^−2^
	SC2018_105mz_19.9073 min	Cyclosativene	2.343	−0.557	0.16 × 10^−2^
	SC2189_156mz_21.5217 min	2,6-Dimethylnaphthalene	2.331	−0.553	0.17 × 10^−2^
	SC2166_93mz_21.2994 min	α-Barbatene	2.225	−0.531	0.31 × 10^−2^
	SC2249_121mz_22.0815 min	β-Alaskene	2.177	−0.508	0.39 × 10^−2^
	SC3261_178mz_31.6228 min	Anthracene	1.818	−0.538	1.90 × 10^−2^
	SC835_57mz_8.7575 min	2-Ethyl-1-hexanol	1.656	−0.540	3.42 × 10^−2^
	SC1762_142mz_17.4948 min	2-Methylnaphthalene	1.582	−0.518	4.40 × 10^−2^
	SC710_43mz_7.5785 min	6-Methyl-5-hepten-2-one	2.458	0.645	0.11 × 10^−2^
***A. thaliana***	SC1858_43mz_18.3994 min	3-Methylbenzamide	1.691	0.731	3.25 × 10^−2^
	SC2131_170mz_20.9725 min	1-Phenoxybenzene	2.329	0.784	2.42 × 10^−2^
	SC2338_43mz_22.9204 min	β-Selinene	1.641	0.672	3.77 × 10^−2^
	SC2775_135mz_27.0408 min	4- *tert* -Octylphenol	2.163	0.549	1.74 × 10^−2^
	SC887_87mz_9.2475 min	Methyl 2-ethylhexanoate	2.631	0.563	0.24 × 10^−2^
	SC1415_120mz_14.2254 min	Methyl salicylate	2.256	−0.594	0.77 × 10^−2^

^1^ In black, compounds strongly correlated with TuYV-infected plants and in grey, compounds strongly correlated with non-infected plants.

## Supplementary Materials

Supplementary Materials can be found at http://www.mdpi.com/1422-0067/19/8/2316/s1.

## Abbreviations

DAS-ELISA	Double-antibody sandwich enzyme-linked immunosorbent assay
GC-MS	Gas chromatography–mass spectrometry
LED	Light-emitted diode
HSSE	Headspace sorptive extraction
PLS-DA	Partial least-square discriminant analysis
VIP	Variable importance in projection
VOC	Volatile organic compounds
