# Supplementary material for: The Aphid-Transmitted Turnip yellows virus Differentially Affects Volatiles Emission and Subsequent Vector Behavior in Two Brassicaceae Plants
Source: Int J Mol Sci. 2018 Aug 7;19(8):2316. doi: 10.3390/ijms19082316 (PMC6121887; doi:10.3390/ijms19082316)
Supplement: Supplementary file 1 [file ijms-19-02316-s001.zip › ijms-317326-supplementary.pdf]

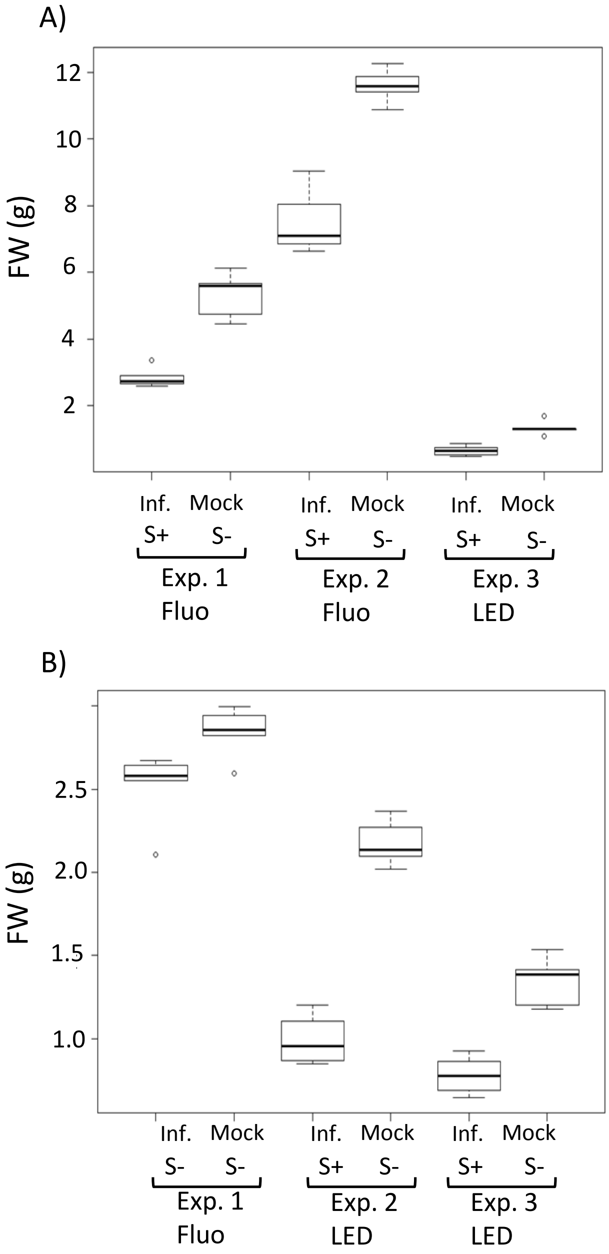

Figure S1: Fresh weight and symptoms of plants used for volatiles collection. Above ground mass of individual fresh plants of A) *C. sativa* and B) *A. thaliana*, 3 weeks after inoculation (except for Exp.3 where *C. sativa* was inoculated two weeks after sowing) with the virus or mock-inoculated (Mock). Results from independent experiments performed in different environmental conditions. Symptom expression (S+: reddening on *A. thaliana* or yellowing on *C. sativa*; S-: no symptom). The light source used to grow plants is indicated (Fluo = cool-white fluorescent lights and LED = Light-emitted diode lamps). Each experiment corresponds to five replicates of 2 plants of *C. sativa* (=10 plants in total per experiment) or 7 plants of *A. thaliana* (=35 plants in total per experiment). FW: fresh weight; Inf.: infected plants controlled by DAS-ELISA.

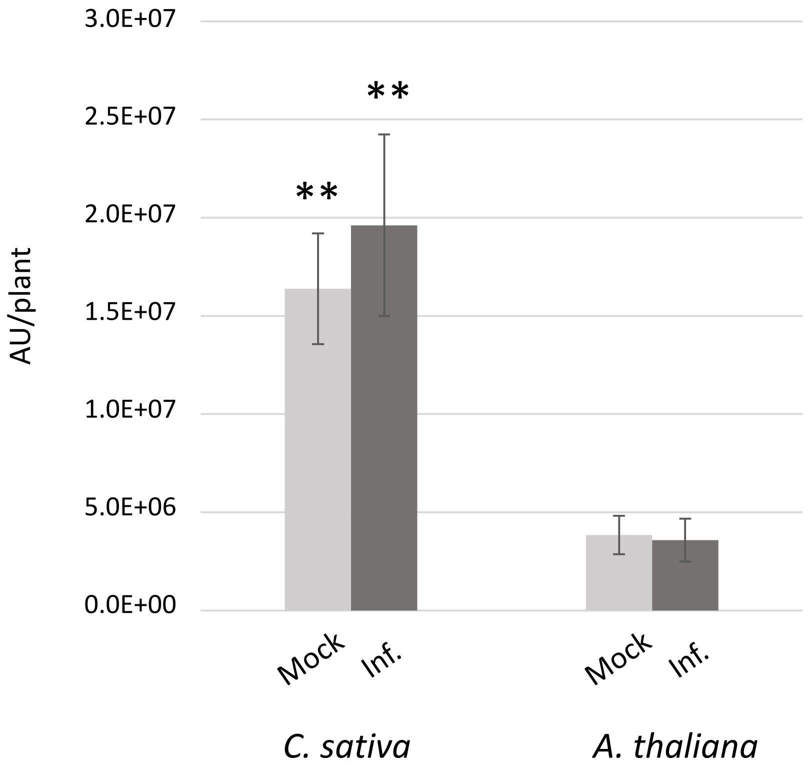

Figure S2: Impact of TuYV infection on global emission of volatiles per plant. Bars (black for TuYV-infected plants and grey for mock-inoculated plants) represent the mean total chromatogram area ± SE (3 experiments with n=5 replicates of 2 plants for *C. sativa*; 3 experiments with n=5 replicates of 7 plants for *A. thaliana*), expressed in area units (AU) per plant. Statistical significance of differences was tested using the Wilcoxon test and compared emission of volatiles between mock-inoculated *C. sativa* and *A. thaliana* and TuYV-infected *C. sativa* and *A. thaliana*. ** = p-value ≤ 0.01.

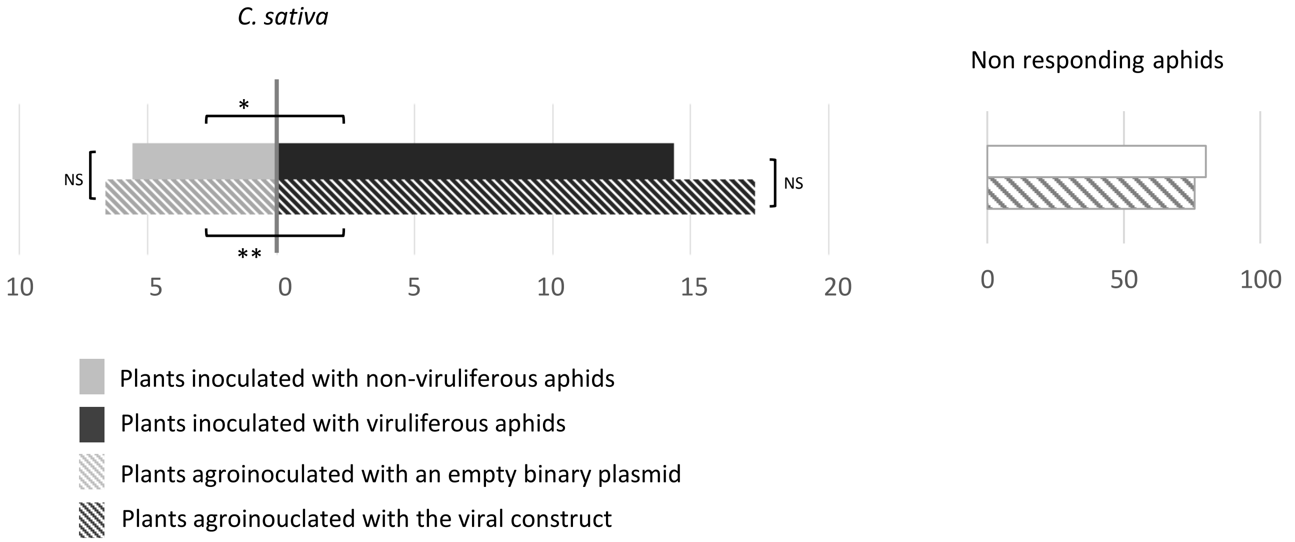

Figure S3: Dual-choice tests using *M. persicae* and plants inoculated with TuYV by two different methods. *C. sativa* plants were either inoculated with viruliferous or non-viruliferous aphids or alternately agroinfiltrated with an empty binary plasmid or the plasmid containing the viral sequence. Non-infected plants (grey bars) and TuYV-infected plants (black bars) were tested versus the opposite modality. Dashed bars stand for agroinoculated plants. The bars on the left represent the percentage of responding aphids that were arrested under a plant. The bars on the right represent the percentage of non-responding aphids still walking in the arena. Results represents 15 repeats of each treatment.

The effect of the virus-inoculation method on *M. persicae* orientation was analysed using GLM with quasi-Poisson distribution (link:log) followed by pairwise comparisons using least-squares means (package R: 'lsmeans'). All statistical analyses were performed in R software.32.

Asterisks indicate a significant difference (* p-value <0.05; ** p-value <0.01) in the distribution of aphids between the two sides of the darkened arena. NS, not significant.

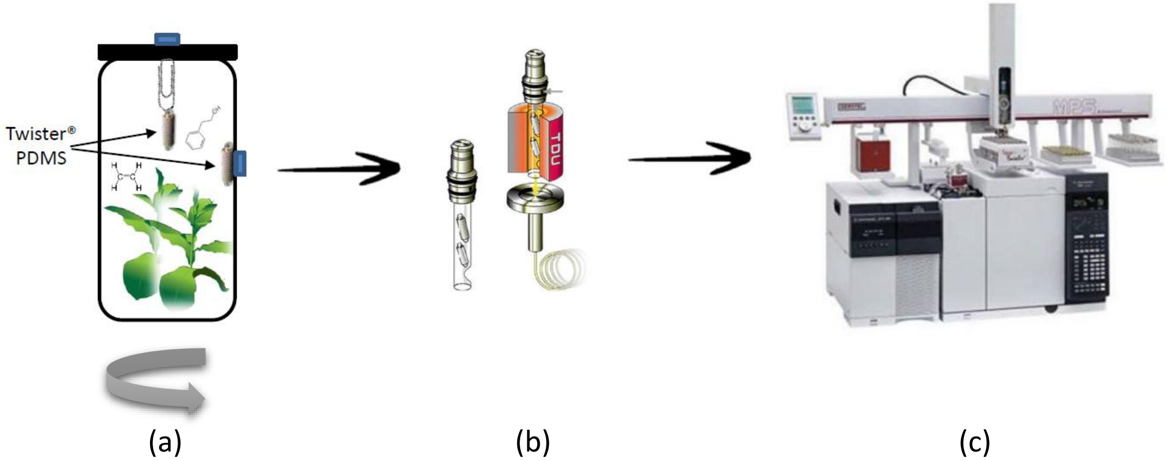

Figure S4: Volatiles collection by Headspace sorptive extraction (HSSE) and analysis by gas-chromatography mass spectrometry (GC-MS). (a) Plants were placed into a 1L glass jar on a shaker and volatiles were collected for 24h on two Twister® positioned inside the jar. The bleu rectangles represent magnets to fix the Twister®. (b) The Twister® are placed together in a glass desorption liner to be thermally desorbed. (c) Metabolite compounds were analyzed by GC-MS.
